# The Profile of Emotional Competence (PEC): Development and Validation of a Self-Reported Measure that Fits Dimensions of Emotional Competence Theory

**DOI:** 10.1371/journal.pone.0062635

**Published:** 2013-05-06

**Authors:** Sophie Brasseur, Jacques Grégoire, Romain Bourdu, Moïra Mikolajczak

**Affiliations:** 1 Department of Education and Technology, University of Namur, Namur, Belgium; 2 Department of Psychology, Université catholique de Louvain, Louvain-la-Neuve, Belgium; 3 Ecole de Psychologues Praticiens de Paris, Université Catholique de Paris, Paris, France; Universidad Europea de Madrid, Spain

## Abstract

Emotional Competence (EC), which refers to individual differences in the identification, understanding, expression, regulation and use of one’s own emotions and those of others, has been found to be an important predictor of individuals’ adaptation to their environment. Higher EC is associated with greater happiness, better mental and physical health, more satisfying social and marital relationships and greater occupational success. While it is well-known that EC (as a whole) predicts a number of important outcomes, it is unclear so far which specific competency(ies) participate(s) in a given outcome. This is because no measure of EC distinctly measures each of the five core emotional competences, separately for one’s own and others’ emotions. This lack of information is problematic both theoretically (we do not understand the processes at stake) and practically (we cannot develop customized interventions). This paper aims to address this issue. We developed and validated in four steps a complete (albeit short: 50 items) self-reported measure of EC: the Profile of Emotional Competence. Analyses performed on a representative sample of 5676 subjects revealed promising psychometric properties. The internal consistency of scales and subscales alike was satisfying, factorial structure was as expected, and concurrent/discriminant validity was good.

## Introduction

Over the last two decades, Emotional Competence (EC) has received increasing attention both from the general public and the scientific community. Sometimes better known as Emotional Intelligence (EI), this concept refers to how individuals deal with intrapersonal or interpersonal emotional information [1]. More specifically, it refers to how an individual identifies, expresses, understands, regulates and uses his emotions or those of others [2], [3]. Note that we prefer the term « Emotional Competence (EC) » to « Emotional intelligence (EI) » because, it is consistent with recent results [4], [5] that show that these competences can be taught and learned (unlike intelligence).

Emotion-related individual differences have been conceptualized as abilities [6], traits [7] or a mix of both [8]. This has led to different important lines of research and to some debates on the status of emotion-related individual differences as being traits (best assessed via personality-like tests) or abilities (best assessed via intelligence-like tests). These debates between traits and ability conceptions of EI have resulted in proposing a model encompassing 3 levels: knowledge, abilities and traits [3], [9]. The first level –the knowledge level- refers to what people know about emotions. The ability level focuses on what people can do (i.e., their maximal performance), and their ability to apply knowledge in a real situation. For instance, even though many people know that acceptance is an efficient strategy to reduce anxiety, many are simply not able to observe and accept their emotions when they are anxious. The trait level refers to the propensity to behave in a certain way in emotional situations. The focus here is not on what people know or can do, but on what they consistently do: their dispositions (i.e., the typical performance). For instance, some individuals may be able to practice acceptance in an exercise if explicitly asked to do so, while not applying this strategy in their life. As the foregoing illustrations should have made obvious, these three levels of emotion-related individual differences are loosely connected. Empirical evidence for these loose relationships has been provided by Lumley, Gustavson, Partridge & Labouvie-Vief [10], who showed that there were only weak correlations between measures of emotional intelligence operationalized as knowledge, abilities and dispositions, respectively. In other words, knowledge does not always translate into abilities, which, in turn, do not always translate into practice (traits). In the current paper, we focus on the trait level.

A considerable amount of research has made much of the significance of EC: indeed, EC appear to influence the most crucial spheres of life: psychological well-being, physical health, social relationships and professional success. At the psychological level, higher EC is for instance associated with greater self-esteem, well-being and life satisfaction [11], [12], as well as a decreased risk to develop psychological disorders or burn-out [13]. At the physical level, EC is related to better physical health and less symptom reporting (see [14], [15] for a meta-analysis), which is not surprising as EC decreases neuroendocrine reactivity to stress [16] and lowers the likelihood to adopt health-damaging behaviours, such as smoking, excessive drinking and reckless driving [17], [18]. At the social level, higher EC leads to better social and marital relationships [19], [20], [21] and, all things being equal, to a greater likelihood to be chosen as a romantic partner [21]. Workwise, EC has been found to be associated with superior academic achievement [22], [23], (including in gifted individuals [24]) and higher job performance, especially for ― but not limited to ― jobs involving high levels of interpersonal contact, such as service occupations (sales, nursing, call centers,…) (see [25], [26] for meta-analyses). Although parts of the foregoing studies are merely cross-sectional, two recent studies suggest that EC is causally involved in these findings. Nelis et al. [4] and Kotsou et al. [5] showed that improving the level of EC through a brief psychological intervention led to increased well-being, decreased cortisol and somatic complaints, enhanced social relationships and greater employability.

Given these multiple implications, the relevance of efficient and valid tools to measure EC is evident. Several EC measurement tools have undergone in-depth validation (see, for instance: [27], [28], [29], [30] ) and have proven to be very useful in predicting a number of effects, thereby making it easier to understand the significance of EC in psychological, somatic, professional and social adjustment. Nevertheless, the further the studies progress, the more it appears important, beyond the relationships revealed, to carefully examine the components involved in these different processes. It can be assumed that *all* the EC do not participate in *all* the effects. Moreover, we can postulate that in some cases, intrapersonal EC carry more weight than interpersonal EC (e.g., predicting health) whereas the opposite would be true in other cases (e.g. predicting the quality of social relationships). However this remains an assumption as to date, no tool is capable of separately measuring the different competencies, separately in self and in others. In addition to the theoretical interest to better understand which competency(ies) participate(s) in which outcomes [15], this interest is equally reflected at the practical level. Recent studies [4], [5] have shown that it is possible to develop one’s EC, even as an adult. The availability, at the clinical level, of a tool that sets out in detail the EC profile of an individual in order to determine towards what goal to work seems therefore relevant.

For these reasons, it seems pertinent to develop a tool that can separately measure the different theoretical dimensions of the construct. This research was conducted to this end. In this paper, we will present the four stages of the development and psychometric validation of the questionnaire: (1) item generation, (2) item reduction, (3) assessment of basic psychometrical properties (reliability, factor structure, norms establishment) (4) assessment of divergent, concurrent and predictive validity. Six samples were collected in total to do so. We assessed basic psychometrical properties using two broad samples. In order to further test the validity of this instrument, the PEC was later included in a study on migraines and emotions in order to examine criterion (concurrent and predictive) validity vis-à-vis trait positive and negative affectivity, and migraine frequency. It was also included in a study on charity seasonal workers to examine objective criterion validity vis-à-vis hierarchical status and fund raising performance. We then recruited a fifth sample to examine the discriminant validity of the scale. We chose to test it vis-à-vis general cognitive ability as studies have repeatedly shown that there were no correlation between cognitive ability and EC (e.g. [15], [31], [28], [30]). Finally, the PEC was also included in a sixth study examining EC in gifted students, in order to test its convergent validity with a measure of trait emotional intelligence.

## Methods

### Participants

In accordance with the ethics code of American psychological association, participants of our research were volunteers and gave their informed consent. Data were treated anonymously. In addition to approximately fifty subjects who were asked to comment on the very first draft of the items, we recruited five samples for a total of 5676 subjects (4753 women and 923 men, aged 15 to 84 years) for this research. Participants of sample 1 (N = 675, mean age = 27.8 SD = 15.67) were recruited among first year Psychology students (53%) and via snowball sampling launched from the first author’s acquaintances (47%). They participated in the item reduction stage. Participants of sample 2 (N = 4306; mean age = 40.61 SD = 13.77) were recruited via the website of a TV broadcast on happiness. They allowed us to examine scale reliability, factor structure, correlations with demographics, and basic criterion validity. Participants of sample 3 (N = 429; mean age = 40.86, SD = 12.86) were recruited among migraine sufferers in order to test validity regarding more specific emotional criteria. Participants of sample 4 (N = 86; mean age = 23, SD = 3.9) were recruited among charity employees to examine validity regarding job-related criteria. Participants of sample 5 (N = 50; mean age = 22.55, SD = 1.96) were recruited among students in their last year of psychology, and served to test discriminant validity. Participants of sample 6 (N = 44, mean age = 16,52 SD = 1,34) were recruited among gifted adolescents to test convergent validity with another self-reported measure of emotional competence.

### Procedure

#### Procedure for item generation

As discussed in the introduction, the test items were designed following the Emotional Competency model developed by Mikolajczak et al. [31]. This model simply replicates the 4 dimensions proposed by Mayer and Salovey but separates the identification from the expression of emotions based on the fact that studies on alexithymia have shown that these dimensions are factorially and conceptually distinct [32]. This model further distinguishes the intrapersonal from the interpersonal aspect of each dimension. 5 to 10 items were therefore constructed for each aspect, for each of the 5 competencies: *identifying* (i.e. being able to perceive an emotion when it appears and identify it), *expressing* (i.e. being able to express emotions in a socially accepted manner), *understanding* (i.e. being able to understand the causes and consequences of emotions, and to distinguish triggering factors from causes), *regulating* (i.e. being able to regulate stress or emotions when they are not appropriate to the context) and *using* (i.e. being able to use emotions to improve reflection, decisions and actions). We generated items for each subscale based on its theoretical definition. As we did not want to create items that would be too redundant, we stopped generating items when they were becoming so (after 7, usually). Some items were inspired by measures like the TEIQue or the SEI (e.g. SEI-R [27]; TEIQue, [7], [33]). In total, 70 items (of which approximately half were inverted) were created and submitted to 50 individuals in order to verify their understandability and their clarity. Several items were reformulated following the feedback received.

#### Procedure for item reduction

The initial questionnaire (70 items) was then completed by 675 persons via internet (Sample 1). Following the web link to the questionnaire, subjects first accessed an introduction page informing them of the research objectives, the voluntary and anonymous nature of their participation and their right to stop it at any time. They provided their consent to participate by clicking to access to the questionnaire per se. For each question, the respondents had to position themselves on a 5-point Likert scale ranging from strongly disagree to strongly agree. In the event that they did not understand the question, they could also tick a box. The items were first analyzed on the basis of their ease of understandability. We had decided to exclude any item misunderstood by more than 5% of the participants. No item was excluded on the basis of this criterion. Items were therefore excluded on the basis of their poor psychometric quality; this was determined by an exploratory analysis of the items and the internal consistency of the subscales. Items with low discrimination indices and strikingly abnormal distributions or items poorly correlated with those belonging to the same group were excluded. We carried out a selection among the remaining items in order to obtain the most consistent scales and eliminate highly redundant items. Based on our analysis, we identified a problem at the regulating of emotions subscale level as the items did not form a coherent whole. Several items were therefore re-written and resubmitted to 50 persons from our sample. The procedure outlined above was applied to these items in order to obtain a coherent subscale. This item reduction phase resulted in a 50-item questionnaire (5 items for each subscale, with 20 reversed items–2 or 3 in each subscale except for the scale “utilization of others’ emotions”). After reversing items, scores for the 5 items of each subscale were averaged to give the score of the subscale. Factor scores were obtained by averaging subscales scores, and the final global score was obtained by averaging the two factor scores.

#### Procedures for assessment of reliability and external validity, validation of questionnaire’s factor structure and norms establishment

The revised 50-item questionnaire (see Appendix S1) was then submitted to 4306 persons (sample 2). In addition to the measure of emotional competence, this internet-based survey included measures of happiness, subjective health and quality of social relationships. It sought to assess criterion (concurrent) validity and included measures of sex, age and professional status in order to examine correlations with demographic variables and establish norms. As explained in the introduction, the final version of the questionnaire has also been included in four other studies (samples 3, 4, 5 and 6) in order to examine concurrent, predictive and divergent validity.

### Measures

***Professional status*** was measured via a “multiple choice” item. Participants were invited to indicate which category, among the 7 following categories, they belonged to: worker (n = 43), skilled worker (n = 69), employed (n = 1489), middle manager (n = 825), senior manager (n = 251), independent (n = 385), unemployed (n = 1117).

***Happiness*** was assessed using the Subjective Happiness Scale (SHS; [34]). The measure comprised 4 items scored on a 7-point Likert scale (sample items were: *I generally consider myself as*… (responses ranged from: totally unhappy to totally happy); *Some people are very happy in general: They enjoy life no matter what happens, making the most of every situation…* (responses ranged from: this sentence doesn’t apply to me to this sentence totally applies to me). It provided a general assessment of whether one is a happy or an unhappy person. The internal consistency in our sample was good (α = .80).

***Subjective health*** was measured by transforming the Subjective Happiness Scale into a Subjective Health scale (on account of the need to have the shortest possible measure). Concretely, the term “happy” was replaced by “in good health” and the term “unhappy” by “in bad health” (e.g. *Compared to most of my relationships, I consider myself*… (responses ranged from: in much poorer health to much healthier). The internal consistency in our sample was good (α = .85).

***Perceived Quality of relationships*** was assessed using a short adaptation of the Quality of Interpersonal Relationships Scale (EQRI; [35]), which measured the quality of the participant’s relationships with their close relatives. This measure consisted of 4 items scored on a 7-point scale (e.g. *I have frequent conflicts with my close relatives* (responses ranged from: do not agree at all to entirely agree). The internal consistency of the measure was.80.

***Trait Positive Emotions*** were measured using 8 items rated on a 5-point scale (ranging from “*never*” to “*very often*”): amazed, relaxed, enthousiastic, easygoing, serene, happy, joyful and appeased, representing high and low arousal emotions, respectively. The internal consistency of the scale was excellent (α = .89).

***Trait Negative Emotions*** were evaluated using 21 items rated on a 5-point scale (ranging from “*never*” to “*very often*”), representing low and high arousal levels of the most common negative emotions (anger, fear, sadness, shame, guilt, frustration, disgust). The internal consistency of the scale was excellent (α = .92).

***Hierarchical status*** of charity seasonal workers ranged from 1 to 5. 1 = fund-raiser (one or two one-month mission in the streets); 2 = confirmed fund-raiser (more than two missions in the streets); 3 = fund-raiser booster (more than two missions & outstanding performance); 4 = team manager; 5 = confirmed team manager. The hierarchical status reflects both the experience and the capacity of the employee. In the present sample, there was a correlation of.44 (p<0.001) between the hierarchical status and the number of missions and of.54 (p<0.001) between the hierarchical status and objective job performance (for statuses 1–4, because people with status 5 are no longer in the streets).

***Job performance*** was assessed via the charity organization’s individual performance indicator (letter from A to E), which reflects the average number of fully fulfilled donation bulletins (signed order for bank transfer) that the employee gathers per hour by approaching people in the streets. These letters correspond to the following performance scores: A = above 0,60; B = 0,55–0,59; C = 0,50–0,549; D = 0,40–0,49 and E = less than 0,399). Because the intervals were not constant, letters were converted to numbers as follows A → 0,62; B → 0.577; C → 0.525; D → 0.45; E→ 0.375. Specifically, we took the mid-point of the interval for B, C and D. For A we took the mid-point of the interval 0.60–0.65 as we had learnt from the organization’s HR manager that performances above.65 were extremely rare. For E, we took the mid-point of the interval 0.035–0.399 as we had learnt that individuals whose performance was lower than 0.35 during the first week were dismissed. Note that the individual performance indicator is only available for people who are still working in the streets (status 1–4), that is, 72 individuals.

***General cognitive ability*** was evaluated by means of the revised version of the *Standard Progressive Matrices* [36], which is one of the most robust predictors of the supra-ordinate factor “g”. This test consists of 50 problem-series and is independent from language and formal schooling. Each problem consists of 4 to 9 figures (arranged as a square) with a missing piece. Below the problem are 6 to 8 alternative pieces to complete the figure, only one of which is correct. Each set involves a different principle for obtaining the missing piece and problems are roughly arranged in increasing order of difficulty. The test was proposed with limited passation time (20 min). It was scored using norms for Belgian population.

***Trait emotional intelligence*** was evaluated by the short form of the Trait emotional intelligence questionnaire (TEIQue-SF; [37]). This measure consists of 30 items scored on a 7-point scale (e.g. *Expressing my emotions with words is not a problem for me* or *I usually find it difficult to regulate my emotions.*). The internal consistency of the scale was satisfying (α = .74).

## Results

### Questionnaire’s Factorial Structure

As the questionnaire was based on a theoretical model, its conceptual validity was verified using factorial analysis. We sought to examine whether, besides the 10 scores available for each of the competencies, the combination of the 5 subscales linked to the management of one’s own emotions and the 5 other subscales (linked to the emotions of others) in two macro-competencies (that is, intrapersonal and interpersonal EC, respectively) was a valid structure. The ten subscales were introduced as target items. Principal axis factoring was selected as the method of extraction (maximum number of iterations was fixed at 25). Because we expected the two factors to be correlated, we selected Oblimin with Kaiser normalization for factor rotation. The number of factors extracted was limited to two in order to determine whether it was reasonable to regroup the items into two factors corresponding to the expected theoretical structure.

Results indicate that the two factors extracted explain 53.53% of the total variance. The KMO index indicated that the factorial solution in two factors was statistically satisfactory (.85). This value indicates that the correlation patterns make it possible to clearly distinguish between the two factors. These are robust, as the factorial saturations in table 1 below show. The utilization dimension is the only one that is subject to moderate saturation in relation to the target factor. Nevertheless, the saturation on the right factor was clearly superior to the secondary saturation on the other factor. All in all, the mean of the saturated main effect is .67; the mean of the secondary saturations is .36. The correlation between the two factors is .47.

**Table 1:** Factorial saturation (oblimin) of the 10 subscales of the PEC (n = 4307).

Subscales		Factor	
		Factor 1	Factor 2
Intrapersonal CE	Identification	.79	.30
	Expression	.76	.45
	Comprehension	.84	.33
	Regulation	.67	.35
	Utilization	.57	.36
Interpersonal CE	Identification	.40	.76
	Expression	.30	.73
	Comprehension	.49	.75
	Regulation	.43	.80
	Utilization	.22	.54

### Internal Consistency

Reliability analysis performed on the 6 samples indicated good internal consistency of the subscales (α from .60 to .83) and a very good consistency of the two factors (> .84) and of the total score (> .88). For illustration purposes, we present below Cronbach’s alpha statistics for one of the samples (the other statistics are available on request). Insofar as the alpha is partially dependent on the sample size, it would have been unrepresentative to present the alphas obtained on the second (overestimated due to a large sample size) and the fourth (underestimated due its small size) sample. Table 2 therefore presents the alphas obtained on the stratified subscale extracted from sample 2 in order to establish the norms (see next section).

**Table 2:** Means, standard deviations and internal consistencies of the PEC subscales and factors for men and woman.

		Male (*n* = 404)		Female (*n* = 405)			Gender differences
Subscales		Mean	SD	Mean	SD	α	t-test significance
Intrapersonal CE	Identification	3.58	.80	3.67	.75	.72	*p = .128*
	Expression	2.93	.88	3.14	.86	.73	*p = .001*
	Comprehension	3.26	.87	3.29	.89	.79	*p = .645*
	Regulation	3.01	.92	2.78	.84	.78	*p = .000*
	Utilization	3.62	.75	3.77	.81	.79	*p = 004*
Interpersonal CE	Identification	3.46	.82	3.65	.77	.83	*p = .001*
	Expression	3.58	.83	3.93	.68	.73	*p = .000*
	Comprehension	3.51	.72	3.71	.66	.77	*p = .000*
	Regulation	3.27	.75	3.42	.67	.79	*p = .002*
	Utilization	3.07	.84	2.87	.83	.81	*p = .001*
Factors scores	Intrapersonal CE	3.28	.62	3.33	.62	.90	*p = .255*
	Interpersonal CE	3.38	.58	3.52	.51	.90	*p = .000*
Global score	CE Global score	3.33	.54	3.42	.49	.93	*p = .008*

### Correlations between Subscales, Factors Scores and Total Score

Bilateral Pearson’s correlations were performed on the 10 subscales, the 2 factors scores and the global score. All correlations are significant (p<.001). Results (Table 3) show strong correlations between each subscale and the global score (from .50 to .71). At the intra-personal level, correlations between subscales are moderate to strong (from .34 to .60). At the inter-personal level, correlations within inter-personal scales are moderate to strong (from .44 to .48), except for the utilization scale which shows lower correlations with other scales (.19 to .41).

**Table 3:** Correlations between PEC subscales, factor score and total score (n = 909).

		Intrapersonal EC						Interpersonal EC					
		Ident.	Exp.	Com.	Reg.	Uti.	Factor score	Ident.	Exp.	Com.	Reg.	Uti	Factor score
Intrapersonal CE	Ident.	1	.50**	.60**	.34**	.33**	.75**	.29**	.34**	.29**	.22**	.27**	.40**
	Exp.	.50**	1	.50**	.42**	.41**.	.78**	.21**	.28**	.24**	.20**	.24**	.34**
	Com.	.60**	.50**	1	.46**	34**	.79**	.26**	.42**	.36**	.27**	.36**	.46**
	Reg.	.34**	.42**	.46**	1	.36**	.71**	.26**	.38**	.28**	.40**	.34**	.48**.
	Uti.	.33**	.41**	.34**	.36**	1	.61**	.16**	.20**	.15**	.27**	.25**	.29**
	Factor score	.75**	.78**	.79**	.71**	.61**	1	.33**	.45**	.36**	.38**	.40**	.54**
Interpersonal CE	Ident.	.29**	.34**	.29**	.22**	.27**	.40**	1	.43**	.58**	.46**	.27**	.76**
	Exp.	.21**	.28**	.24**	.20**	.24**	.34**	.43**	1	.47**	.54**	.19**	.72**
	Com.	.26**	.42**	.36**	.27**	.36**	.46**	.58**	.47**	1	.44**	.23**	.74**
	Reg.	.26**	.38**	.28**	.40**	.34**	.48**.	.46**	.54**	.44**	1	.41**	.78**
	Uti.	.16**	.20**	.15**	.27**	.25**	.29**	.27**	.19**	.23**	.41**	1	.60**
	Factor score	.33**	.45**	.36**	.38**	.40**	.54**	.76**	.72**	.74**	.78**	.60**	1
CE	Global score	.63**	.71**	.67**	.63**	.61**	.89**	.65**	.60**	.67**	.71**	.50**	.86**

*Note.* PEC = Profile of Emotional Competence.EC = Emotional Competence. Ident.: Identification. Exp.: Expression. Comp: Comprehension. Reg.: Regulation. Uti.: Utilization.

* *p<.*05

** *p<.*001.

### Means and SD’s (Norms)

The norms were established on the basis of a stratified subscale selected among the 4306 initial subjects. The objective of this stratification was to balance gender as well as participants’ ages. We also took into account the socio-professional category in order to match as closely as possible the distribution in the general population (INSE norm for the French population in 2010). Owing to the significant differences in gender, Table 2 presents the mean and the standard deviation for each gender separately.

### Relationship with Demographic Variables

We then tested the relationships between the three global EC scores (intrapersonal EC, interpersonal EC and global EC) and the 3 demographic variables: age, sex and socio-professional status. In order to take into account the covariance between the predictors, we performed a multivariate analysis of variance (MANOVA). The measure used was the Pillai’s trace.

#### Gender

MANOVAs refine the mean comparisons (t-tests) reported in Table 2: There was no significant effect of gender on the global EC score (V = .001, F (2.4087) = 2.19, *p>.05),* or on the intra-personal score, but there was a significant effect on the interpersonal EC score, F (1.4088) = 3.75, *p = .05*. Gender differences at the subscale level are reported in table 2.

#### Age

Simple correlations between EC and age figure in Table 4. In order to run MANOVAs (which require categorical predictors), we created 7 age groups. There was a significant main effect of age on EC, V = .006, *F (14.8176) = *1.88, *p<05.* Results were respectively *F (7.4088) = *2.40, *p<.05* for the intrapersonal score*; F (7.4088) = *2.81, *p<.05* for the interpersonal score and*; F (7.4088) = *2.32, *p<.05* for the global score. Tuckey *post hoc* tests revealed that people under 25 scored lower than people between 26 and 50, who scored lower than people above 51 years old.

**Table 4:** Correlations of the PEC subscales and factors with age and indicators of convergent, divergent and concurrent validity.

		Intrapersonal EC								Interpersonal EC							
	Sample size	Ident.	Exp.	Com.	Reg.		Uti.	Factor score		Ident.	Exp.	Com.	Reg.	Uti.	Factor score	Global score	
Demographics																	
Age	4307	.15**	.12**	.18**	.13**		.01	.17**	−.01		.09**	.04**	.12**	−.12**	.03	.12**	
Discriminant validity																	
Cognitive ability	49	.03	.09	.07	.06		.16	.11	.11		.24	.20	.23	.21	.28	.23	
Convergent validity: TEIQue-SF)	44	.60**	.60**	.60**	.54**		.37*	.78**	.50**		.27	.55**	.24	.03	.52**	.77**	
Criterion validity																	
Happiness	4307	.25**	.33**	.30**	.46**	.28**		.44**		.14**	.11**	.19**	.24**	.19**	.24**	.40**	
Subjective health	4201	.12**	.10**	.14**	.20**	.10**		.18**		.04**	.03**	.04**	.08**	.08**	.08**	.15**	
Quality of social relationship	4196	.31**	.41**	.33**	.28**	.28**		.47**		.22**	.22**	.28**	.33**	.25**	.36**	.48**	
Positive affectivity	429	.31**	.36**	.38**	.53**	.20**		.51**		.19**	.17**	.25**	.26**	.19**	.29**	.46**	
Joy	429	.29**	.31**	.34**	.33**	.44**		.48**		.44**	.18**	.18**	.24**	.26**	.29**	.39**	
Relaxation	429	.22**	.26**	.31**	.37**	.54**		.46**		.39**	.15**	.14**	.21**	.22**	.23**	.44**	
Negative affectivity	429	−.31**	−.38**	−.37**	−.53**	−.07		−.49**		−.14**	−17**	−.25**	−.20**	−.11**	−.23**	−.41**	
Anger	429	−.17	−.26**	−.22**	−.38**	.01		−.31**		−.12**	−.19**	−.21**	−.19**	−.02	−.19**	−.27**	
Fear	429	−.21**	−.28**	−.39**	−.52**	−.13**		−.45**		−.09	−.08	−.18**	−.19**	−.11*	−.17**	−.36**	
Sadness	429	−.30**	−.32**	−.35**	−.51**	−.09		−.46**		−.10**	−.11*	−.21**	−.16**	−.13**	−.19**	−.37**	
Guilt	429	−.27**	−.28**	−.29**	−.36**	−.01		−.36**		−.08	−.11*	−.15**	−.14**	−.14**	−.17**	−.30**	
Disgust	429	−.18**	−.27**	−.21**	−.24**	−.04		−.28**		−.14**	−.14**	−.19**	−.11**	−.02	−.16**	−.25**	
Frustration	429	−.28**	−.33**	−.26**	−.40**	−.04		−.39**		−.12*	−.14**	−.21**	−.13**	−.87	−.19**	−.33**	
Hierarchical status	86	.38**	.06	.37**	.21*	.35**		.43**		.20*	.02	.10	.15	.40**	.27*	.46**	
Job performance	72	.15	−.04	.23*	−.02	.06		0.09		.29*	.11	.07	−.01	.28*	.19*	.22*	

*Note.* PEC = Profile of Emotional Competence. Ident.: Identification. Exp.: Expression. Comp: Comprehension. Reg.: Regulation. Uti.: Utilization. TEIQue-SF = Trait emotional intelligence questionnaire short form.

* *p<.*05

** *p<.*001.

#### Professional status

There was a significant main effect of socio-professional category on EC, V = .008, *F (12.8176) = *2.90, *p = .001* on both intra (*F* (6.4088) = 2.40, *p<.05*) and interpersonal EC scores (*F* (6.4088) = 3.89, *p = .001)*. As shown by the Tuckey *post hoc* test, freelance workers have the highest level of EC (significantly higher than all others except senior managers). Senior managers come next (scoring significantly higher than all the others except skilled workers). Skilled workers, employed, middle managers and unemployed follow in this order, without differing statistically from each other. Workers have significantly lower scores than all other occupational categories.

### Convergent Validity

Convergent validity was assessed by examining Pearson correlations between PEC and TEIQue-SF scores. As expected and as shown in table 4, PEC and TEIQue-SF scores are significantly correlated. The association with the TEIQue-SF is strong for the PEC global and intrapersonal factor scores, and moderate for the interpersonal factor score. At subscales levels, some interpersonal subscales do not correlate with TEIQue’s global score, thereby highlighting significant differences between instruments despite their convergent validity.

### Concurrent Validity

Criterion validity was assessed by examining Pearson correlations between EC global and factor scores with happiness, subjective health, perceived quality of social relationships, trait positive affect and trait negative affect, hierarchical status and job performance.

As expected, and as shown in table 4, EC is highly associated with happiness. The association is stronger with the intrapersonal score than with the interpersonal score, but both contribute to the correlation. EC is also significantly associated with subjective health (but to a lesser extent), although this association was essentially due to the relation with the intrapersonal EC score. As expected, EC is also highly associated to the quality of social relationships. Both intrapersonal and interpersonal scores contribute to the correlation.

Correlations with positive and negative affectivity also yielded the expected results: EC was a strong predictor of both positive and negative affects. Associations with affective states were logically stronger with intrapersonal EC, but still significant with interpersonal EC. Note that EC (especially intrapersonal EC) was also negatively associated with migraine frequency but we have not reported that here as this is part of another paper (Miliche et al., in preparation).

Finally, EC was also significantly associated with the hierarchical status and job performance of seasonal workers recruiting donations for charity organizations. Both intra-and inter-personal EC participated to the correlation with hierarchical status (which depends on both experience-perseverance- and performance) but only inter-personal EC was associated with job performance (i.e., the number of donators recruited). Correlations at the subscale level showed that, unsurprisingly, the most predictive emotional competencies were the ability to identify and use others’ emotions.

### Divergent Validity with General Cognitive Ability

As expected, neither global EC nor intra or interpersonal EC relate to general cognitive ability (see table 4).

## Discussion

This study sought to develop and validate a measure of EC capable of distinctly measuring the five core emotional competences, separately for one’s own and others’ emotions. The questionnaire encompasses 10 subscales (intrapersonal identification, intrapersonal expression, intrapersonal comprehension, intrapersonal regulation, intrapersonal utilization, interpersonal identification, interpersonal expression, interpersonal comprehension, interpersonal regulation and interpersonal utilization) of 5 items each (with 2 or 3 inverted), grouped into two factors (intrapersonal EC and interpersonal EC) and one global score. This research suggests that the questionnaire has promising psychometric properties. The internal consistency of the 10 subscales is good, especially if we consider the small number of items (5) that compose each subscale. The two global scores (intrapersonal EC and interpersonal EC), as well as the total score also show good internal consistency. Moreover, the factorial analysis performed confirms the validity of the calculated scores, and the relevant subscales all present satisfactory factorial saturation in regard to the theoretically determined factor. At the theoretical level, these results confirm both the link between the intra- and inter-personal dimensions of EC, as well as their relative independence. They support the relevance of assessing both dimensions, separately. The usefulness of distinguishing these two dimensions is illustrated in a recent study on gifted students by Brasseur & Grégoire (in preparation). The use of the PEC permitted to go deeper into previous results obtained by the same team and others [38] and, specifically, to show a specificity in the EC profile of gifted students: Compared to controls, they have lower intra-personal EC and higher inter-personal EC (while they have difficulties in identifying, expressing and understanding their emotions, they are particularly good at regulating and using others’ emotions). These findings, which nicely corroborate clinical observations, are interesting both theoretically (in showing possible asymmetries in the development of intra- and inter-personal EC) and practically (in suggesting areas of improvement).

As regards the influence of demographic variables on EC, our results replicate previous studies in this field. The positive correlation that we identified between EC and age is consistent with the results of other studies [39]. Interestingly, the present study suggests that this correlation is particularly due to an improvement in intra-personal competencies, with inter-personal competencies appearing to be proportionally less sensitive to age. Future studies should pursue these findings. As concerns gender, while t-test suggested at first sight a significant difference in global EC in favour of women, MANOVA do not confirm this difference. Thus, EC are not reserved exclusively to women, a result that did not prevent us from observing a significant difference between men and women on several competencies. For instance, women score higher on emotion expression while men score higher on emotion regulation. At the intrapersonal level, these differences counterbalance each other in such a way that gender differences on the total intrapersonal score are not significant. At the interpersonal level, men make better use of the emotions of others than women, but the latter have higher scores than men on all the other dimensions (identifying, understanding, listening, regulating others’ emotions), results that give them a slight advantage as regards interpersonal EC. This is consistent with other research findings (e.g. [40]) or public observations (e.g. [41]), which highlight the tendency for women to develop listening competence, empathy competence and to be more attentive to non verbal signals. Better scores among women as regards expressing their emotions is in line with their social roles, emotional expression being generally thought unwelcome among men (“men do not cry”). This can also explain why women have better scores in all that relates to interpersonal EC as they are socially encouraged to take care of others and to share at an emotional level. Significantly higher scores among men as regards regulating their own emotions is consistent with previous measurements (e.g. better scores in EQi Stress management [42]) and with the male socialisation of emotions (“men must be strong”). Lastly, the fact that men appear to perform better as regards using the emotions of others is consistent with Kray & Thompson’s [43] results which show that men are more inclined to use the emotions of others in order to influence their decisions.

As far as convergent validity is concerned, the global PEC score was highly correlated with the TEIQue thereby showing evidence of convergent validity with a widely used measure of emotional competence. The intra-personal factor (and subscales) of the PEC correlated more than its inter-personal factor (and subscales) with the TEIQue, which was expected as the TEIQue encompasses more intra- than inter-personal dimensions. Taken together, the pattern of correlations suggests that these instruments cannot be reduced to one another, despite evidence of convergent validity.

As far as concurrent validity is concerned, PEC scores are, as expected, associated with greater happiness, better subjective health, better social relationships, greater positive affectivity and lower negative affectivity [11], [13], [19], [21]. In addition, they were also able to predict objective criteria such as hierarchical status and performance in a job requiring high EC. Interestingly, the subscales that were the most predictive of performance (i.e. identification and use of others’ emotions) fit well with what a seasonal worker told us after the study “*We are not alchemists, we are not here to turn lead into gold, we are gold seekers, we are here to find individuals who want to take action and to help them make the necessary steps*.” To be good at this, you must be able to quickly identify how the passer-by feels, you do not want to lose time with people who are unlikely to give and to convert their emotion in a donation. Consistent with others [17], [28], [33], we found no relation between EC and IQ.

Taken together, and although future studies will have to supplement validity analyses (e.g. with 360° measurements or biological measurements), the foregoing results suggest that the tool’s psychometric properties are promising. Despite the fact that other measures are advantageous as they are shorter (EIS, [30]; EIS-R, [27]; TEIQUE-SF, [44]) and can be privileged when the only objective is to obtain a global EC score, the PEC represents added value when the objective is to obtain a detailed profile of emotional competencies for research and/or clinical purposes. In this respect, it offers some interesting possibilities. On a strictly clinical level, by making it possible to better identify an individual’s profile, the PEC offers the necessary information to adjust interventions to specific profiles. At the research level, it makes it possible to better identify the processes behind a given outcome. For example, research conducted by Miliche et al. (in preparation) on migraine patients has found that the protective effect of EC vis-à-vis crisis frequency does not involve *all* the EC; only some of them are protective. These results are interesting as they suggest that intervention protocols must focus on these competencies. The PEC also offers interesting possibilities in the management field, making it possible to first highlight the EC that are most necessary for a specific task/job and then adapt the selection and training processes accordingly. In this study, the PEC demonstrates that only the interpersonal EC level, and more specifically, the ability to identify and use others’ emotions, influences the amount of donations received by associations’ fund raisers. The organization has therefore concrete avenues that can be used to improve the selection and performance of its fund raisers. Results like these constitute a good example of what the PEC is made for and of the perspectives it offers. We hope that future studies will soon confirm its utility and strengthen its validity.

## Supporting Information

Appendix S1 **English version of the “PEC” questionnaire.**
(DOCX)
