# Supplementary material for: The Profile of Emotional Competence (PEC): Development and Validation of a Self-Reported Measure that Fits Dimensions of Emotional Competence Theory
Source: PLoS One. 2013 May 6;8(5):e62635. doi: 10.1371/journal.pone.0062635 (PMC3646043; doi:10.1371/journal.pone.0062635)
Supplement: Appendix S1 — English version of the “PEC” questionnaire. (DOCX) [file pone.0062635.s001.docx]

**Appendix S1**

**The Profile of Emotional Competence**

**Note for the readers: items are presented in a random order**

Many thanks for taking the time to answer this questionnaire. We appreciate your interest.

The questions below are designed to provide a better understanding of how you deal with your emotions in daily life. Please answer each question spontaneously, taking into account the way you would normally respond. There are no right or wrong answers as we are all different on this level.

For each question, you will have to give a score on a scale from 1 to 5, with 1 meaning that the statement does not describe you at all or you never respond like this, and 5 meaning that the statement describes you very well or that you experience this particular response very often.

|  | 1 | 2 | 3 | 4 | 5 |
| --- | --- | --- | --- | --- | --- |
| 1. As my emotions arise I don't understand where they come from. |  |  |  |  |  |
| 1. I don't always understand why I respond in the way I do. |  |  |  |  |  |
| 1. If I wanted, I could easily influence other people's emotions to achieve what I want. |  |  |  |  |  |
| 1. I know what to do to win people over to my cause. |  |  |  |  |  |
| 1. I am often a loss to understand other people's emotional responses. |  |  |  |  |  |
| 1. When I feel good, I can easily tell whether it is due to being proud of myself, happy or relaxed. |  |  |  |  |  |
| 1. I can tell whether a person is angry, sad or happy even if they don't talk to me. |  |  |  |  |  |
| 1. I am good at describing my feelings. |  |  |  |  |  |
| 1. I never base my personal life choices on my emotions. |  |  |  |  |  |
| 1. When I am feeling low, I easily make a link between my feelings and a situation that affected me. |  |  |  |  |  |
| 1. I can easily get what I want from others. |  |  |  |  |  |
| 1. I easily manage to calm myself down after a difficult experience. |  |  |  |  |  |
| 1. I can easily explain the emotional responses of the people around me. |  |  |  |  |  |
| 1. Most of the time I understand why people feel the way they do. |  |  |  |  |  |
|  | 1 | 2 | 3 | 4 | 5 |
| 1. When I am sad, I find it easy to cheer myself up. |  |  |  |  |  |
| 1. When I am touched by something, I immediately know what I feel. |  |  |  |  |  |
| 1. If I dislike something, I manage to say so in a calm manner. |  |  |  |  |  |
| 1. I do not understand why the people around me respond the way they do. |  |  |  |  |  |
| 1. When I see someone who is stressed or anxious, I can easily calm them down. |  |  |  |  |  |
| 1. During an argument I do not know whether I am angry or sad. |  |  |  |  |  |
| 1. I use my feelings to improve my choices in life. |  |  |  |  |  |
| 1. I try to learn from difficult situations or emotions. |  |  |  |  |  |
| 1. Other people tend to confide in me about personal issues. |  |  |  |  |  |
| 1. My emotions inform me about changes I should make in my life. |  |  |  |  |  |
| 1. I find it difficult to explain my feelings to others even if I want to. |  |  |  |  |  |
| 1. I don't always understand why I am stressed. |  |  |  |  |  |
| 1. If someone came to me in tears, I would not know what to do. |  |  |  |  |  |
| 1. I find it difficult to listen to people who are complaining. |  |  |  |  |  |
| 1. I often take the wrong attitude to people because I was not aware of their emotional state. |  |  |  |  |  |
| 1. I am good at sensing what others are feeling. |  |  |  |  |  |
| 1. I feel uncomfortable if people tell me about their problems, so I try to avoid it. |  |  |  |  |  |
| 1. I know what to do to motivate people. |  |  |  |  |  |
| 1. I am good at lifting other people's spirits. |  |  |  |  |  |
| 1. I find it difficult to establish a link between a person's response and their personal circumstances. |  |  |  |  |  |
| 1. I am usually able to influence the way other people feel. |  |  |  |  |  |
| 1. If I wanted, I could easily make someone feel uneasy. |  |  |  |  |  |
| 1. I find it difficult to handle my emotions. |  |  |  |  |  |
|  | 1 | 2 | 3 | 4 | 5 |
| 1. The people around me tell me I don't express my feelings openly. |  |  |  |  |  |
| 1. When I am angry, I find it easy to calm myself down. |  |  |  |  |  |
| 1. I am often surprised by people's responses because I was not aware they were in a bad mood. |  |  |  |  |  |
| 1. My feelings help me to focus on what is important to me. |  |  |  |  |  |
| 1. Others don't accept the way I express my emotions. |  |  |  |  |  |
| 1. When I am sad, I often don't know why. |  |  |  |  |  |
| 1. Quite often I am not aware of people's emotional state. |  |  |  |  |  |
| 1. Other people tell me I make a good confidant. |  |  |  |  |  |
| 1. I feel uneasy when other people tell me about something that is difficult for them. |  |  |  |  |  |
| 1. When I am confronted with an angry person, I can easily calm them down. |  |  |  |  |  |
| 1. I am aware of my emotions as soon as they arise. |  |  |  |  |  |
| 1. When I am feeling low, I find it difficult to know exactly what kind of emotion it is I am feeling. |  |  |  |  |  |
| 1. In a stressful situation I usually think in a way that helps me stay calm. |  |  |  |  |  |
